# Factors related to treatment intensity in Swiss primary care

**DOI:** 10.1186/1472-6963-9-49

**Published:** 2009-03-18

**Authors:** André Busato, Pius Matter, Beat Künzi

**Affiliations:** 1 Institute for Evaluative Research in Orthopaedic Surgery, University of Bern, Stauffacherstrasse 78, CH-3014, Bern, Switzerland; 2 University of Bern, Department of Economics, Schanzeneckstrasse 1, CH-3001 Bern, Switzerland; 3 Swisspep – Institute for Quality and Research in Healthcare, Postfach – CH 3073 Guemligen, Switzerland

## Abstract

**Background:**

Questions about the existence of supplier-induced demand emerge repeatedly in discussions about governing Swiss health care. This study therefore aimed to evaluate the interrelationship between structural factors of supply and the volume of services that are provided by primary care physicians in Switzerland.

**Methods:**

The study was designed as a cross-sectional investigation, based on the complete claims data from all Swiss health care insurers for the year 2004, which covered information from 6087 primary care physicians and 4.7 million patients. Utilization-based health service areas were constructed and used as spatial units to analyze effects of density of supply. Hierarchical linear models were applied to analyze the data.

**Results:**

The data showed that, within a service area, a higher density of primary care physicians was associated with higher mortality rates and specialist density but not with treatment intensity in primary care. Higher specialist density was weakly associated with higher mortality rates and with higher treatment intensity density of primary care physicians. Annual physician-level data indicate a disproportionate increase of supplied services irrespective of the size of the number of patients treated during the same year and, even in high volume practices, no rationing but a paradoxical inducement of consultations occurred. The results provide empirical evidence that higher densities of primary care physicians, specialists and the availability of out-patient hospital clinics in a given area are associated with higher volume of supplied services per patient in primary care practices.

Analyses stratified by language regions showed differences that emphasize the effect of the cantonal based (fragmented) governance of Swiss health care.

**Conclusion:**

The study shows high volumes in Swiss primary care and provides evidence that the volume of supply is not driven by medical needs alone. Effects related to the competition for patients between primary care physicians, specialists and out-patient hospital clinics and an association with the system of reimbursing services on a fee-for-service basis can not be excluded.

## Background

Swiss health care is based on the principles of universal health care, granting access to a broad range of services and, to a large extent, the financial protection is achieved through mandatory health insurance coverage. The system is also based on free demand and supply and most ambulatory services are reimbursed on a fee-for-service system. Health care expenditures per capita rank second after the US among OECD countries and are increasing[1]. Swiss health care is fragmented into 26 individually managed cantonal health systems[1,2] including 4 different languages and cultures, which may be seen as a natural laboratory for health services research. National authorities have therefore little empowerment and nationwide reforms aimed to contain health care expenditures are difficult to achieve. As in many other OECD countries, costs are expected to increase further in the near future, as a result of an aging and multimorbid population and high expenses for technological innovations in medicine.

Current cost containment policies promote market-based mechanisms and are focused on higher self-responsibility of everyone involved and on measures to enhance the transparency of the system, e.g. by peer comparison of services provided to limit possibilities of abuse.

The supplier-induced or, more specifically, the physician-induced demand (SID) hypothesis is based on the assumption that physicians influence the demand for their services by controlling the information exchanged with their patients[3]. This may result in situations where patients, who are not highly informed, delegate the decisions about the scope and extent of their treatment to their physicians. Consequently, the quantity of supplied services may not be driven by the medical needs of the patient population alone.

Although the existence of SID has not been adequately established[4], it is often used to legitimize regulative interventions targeting the supply of medical resources. The literature underlines two characteristics that empirically identify SID: High supply and the related competition among providers [5-7]. This study aimed at testing the following SID related hypotheses:

- A higher density of physicians is associated with increasing quantities of services per physician.

- Physicians who treat high numbers of patients on a fee-for-service basis have less contacts, on average, with each patient and vice versa; physicians with a smaller patient populations compensate the lack of patients with additional contacts.

The study analyzes therefore non-medical factors that may influence the number of interactions between physicians and patients. It is part of a national project to evaluate the availability and utilization of resources in Swiss ambulatory health care[8,9]. The focus of this article is limited to primary care and includes data from primary care physicians (PCP) with either board certification of the Swiss Medical Association for general practice/family medicine or general internal medicine as well as general practitioners without board certification.

## Methods

### Data

A database of census data from the Swiss population for 2004 was created within the project. This database included a complete list of billing numbers from all Swiss physicians with ambulatory practices, and the corresponding consultations that were reimbursed by basic health insurers. Physicians were classified into PCP's and specialists based on their last acquired certification issued by the Swiss medical association. Health service areas for primary care were constructed according to Goodman et al[10] and used as the geographical units of analyses (1014 areas in total). A description of the health service area construction and characteristics within Swiss primary health care is detailed in a earlier publication [9]. Health service areas were additionally classified into urban health services areas which consisted only of communities classified as urban centres, suburban- or periurban communities and into rural health service areas which included only communities classified as agricultural-rural or agricultural-mixed. The typology of communities was adopted from the Swiss Federal Statistical Office [9]. Mortality rates of health service areas were calculated based on the death statistics of communities located in each health service area. Census data and death statistics were obtained from the Swiss Federal Statistical Office. The statistical procedures in this study focused on the relationship between the annual number of patients and the respective volume of services that were provided by Swiss PCP's. We defined treatment intensity as the average number of consultations for individual patients of each physician. This measure was obtained by dividing the number of consultations by a physician by the number of patients treated. In this context, the term consultation includes all encounters that were reimbursed by basic health insurance, i.e. face to face consultations, telephone consultations and home visits. Only data from consultations regarding illness and maternity were included. Accident-related consultations were excluded due to different insurance coverage.

### Data analysis

Descriptive analyses using tables and graphs indicated skewed distributions for treatment intensity. Medians and means are therefore given in the results tables in order to show the central tendencies of the data.

Two statistical models were developed to explore the effects that structural factors of care providers and demographic attributes of patient populations had on practice volumes of physicians. These two models were developed based on the method described by Grytten et al[4].

In both models, the relationship between the number of annual consultations of each physician and the number of patients treated in the same year was studied in a log-log manner. This allowed interpretation of the results using the concept of elasticity[4]. In this framework, linear relationships with a unitary slope would indicate proportionality whereas slopes below 1 would indicate that treatment intensities decrease with high annual patient numbers and vice versa. The slope of the regression line, i.e. the location of the supply curve, is thus of central importance in the following analyses. In contrast to other investigations, we used multilevel mixed models to account for the fact that data were available at both health service area and individual physician level[11].

The first model was used to estimate the overall association between the annual number of patients and the respective number of consultations. The second model was based on the assumption that the relationship between patient number and volume of consultations may not be linear. Thus physician level data was divided into four groups (quartiles) of annual patient numbers and each of these groups was studied individually.

The following relationships describe the four groups:

-   P_Q1 =   log(annual patient number) for ≤ 402 patients

   otherwise P_Q1 = 0   (1st Quartile)

-   P_Q2 =   log(annual patient number) for > 402 ≤ 735 patients

   otherwise P_Q2 = 0   (2nd Quartile)

-   P_Q3 =   log(annual patient number) for > 735 ≤ 1079 patients

   otherwise P_Q3 = 0   (3rd Quartile)

-   P_Q4 =   log(annual patient number) for > 1079 patients

   otherwise P_Q4 = 0   (4th Quartile)

An equal set of explanatory factors including structural attributes of care providers and demographic data of the patient population were added to both models. Table 1 provides an overview of the structure of each model.

**Table 1:** Structure of hierarchical linear models

Type of variable	Description
**Outcome**	Number of annual physician consultations

**Explanatory variables**	

- Level I (physician)	Model 1: Number of patients treated in 2004 ^a^.
	Model 2: 4 indicator variables (P_Q1 – P_Q4) representing quartiles of the number of patients treated in 2004^a^
	Physician gender
	Physician age
	Professional qualification^b^
	Average patient age
	Proportion of female patient consultations

- Level II (service area)	Number of PCP's per 10,000 inhabitants
	Number of specialists^c ^per 10,000 inhabitants
	Presence of a hospital providing ambulatory services (coded as a binary variable with 0 for no hospital and 1 for at least one hospital).
	Number of deaths per 1000 inhabitants (mortality)

^a ^See Table 4 for results of model 1 and 2.

^b ^Board certification of the Swiss Medical Association (family medicine, general internal medicine, general practitioners without board certification).

^c ^Physicians providing ambulatory care but not classified as primary care provider (board certification in other medical disciplines)

Previous analyses of treatment incidence in the same data set showed considerable variations between the different language areas and hence cultural regions in Switzerland[9]. The model was therefore additionally run for each language region separately (language stratified Model I). Since there were only two service areas (<1%) associated with the fourth national language (Romansh), these areas were grouped with the Swiss-German areas[9] within these separate analyses.

The descriptive analyses identified a few records with extreme numbers of consultations and/or patients. The data used in this study are based on billing numbers that may be shared by multiple physicians. According to santesuisse (umbrella organisation of Swiss of health insurers), these situations are rare and our descriptive analyses confirmed this. Nevertheless, we considered all patient and consultation numbers above the respective 99th percentiles as outliers and excluded them from further analysis (more than 2283 patients or more than 9742 consultations – 96 records removed) the resulting data pool therefore included 6087 primary care providers.

Variance components of service area level variables quantifying the corresponding proportions of outcome variation accounted for by specific variables were additionally calculated [12]. Residual analyses, performed to validate the statistical procedures, showed no evidence of assumption violations for the models used in the analysis. SAS 9.1 (SAS Institute Inc., Cary, NC, USA) and "proc mixed" were used for all analyses, and the level of significance was set at 0.05 throughout the study.

## Results

### Structure of supply

In 2004, the 6087 primary care physicians analysed treated 4.67 million patients with an average number of patients treated in 2004 of 766.84 and an average number of annual consultations per patient of 4.33. Detailed data on the distribution of physicians, patients and consultations across the four Swiss language regions are given in Table 2 (Additional file 1). The average density of physicians per 10,000 inhabitants within health service areas was 8.01 for primary care physicians and 4.46 for specialists (Additional file 2: Table 3). Considerable differences were observed between urban and rural health service areas, densities of primary care physicians and specialists in urban areas were 7.69 and 5.84 per 10,000 inhabitants vs. 8.38 and 0.86 respectively in rural areas. Hospital departments providing ambulatory care were present in 222 of the 1014 service areas (22%). The localisation index (LI)[13], which quantifies the degree of primary care provided locally for the population in a given area, was also calculated and showed that on average 57% of all required primary care services were used from local physicians.

**Figure 1:**
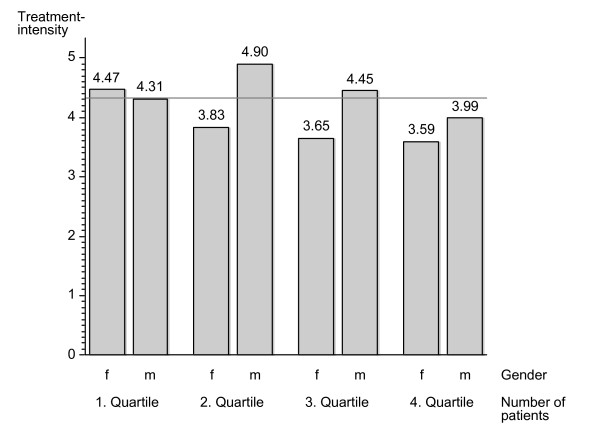
**Graph displaying treatment intensity against number of patients**.

Significant differences were found between female and male physicians. On average, female physicians treated 42% fewer patients and performed 51% fewer consultations than their male peers, resulting in a lower treatment intensity with female physicians (4.1 consultations/year) than with male physicians (4.4 consultations/year). Female physicians performed 67% of their consultations with female patients vs. 53% for male physicians. Differences between male and female physicians were also evident across the four groups of annual patient numbers (P_Q1-Q4) particularly in the two middle groups (Figure 1). In health service areas with ambulatory hospital care average treatment intensity was 4.40 vs. 4.22 in areas without hospitals.

### Relationships between resource availability and scope of services

Spearman correlation coefficients (ρ) were used to explore the linear relationships between various variables (Additional file 2: Table 3). These results showed only minor associations between the age of the physicians and the annual number of patients or the number of consultations but moderate positive associations between the average age of the patients and the treatment intensity. The relationships between resource availability and scope of services per patients were explored at the level of service areas and indicated a small but significant correlation between the density of specialists and the average treatment intensity, whereas a non-significant correlation was found between the density of primary care and average the treatment intensity. Significant correlations were observed between both regional mortality and PCP density (ρ = 0.22), and treatment intensity (ρ = 0.17) and between mortality and specialist density (ρ = 0.072) (Additional file 2: Table 3). Average mortality in urban health service areas was 7.08 deaths per 1000 inhabitants vs. 8.73 in rural areas.

Estimates on the effect of multiple variables on the annual number of consultations are given in Table 4 (Additional file 3). The log-log linear regression of consultations vs. annual number of patients yielded a slope of 1.108 with the 95% confidence interval ranging from 1.10 to 1.12. This indicates a significant non-proportional relationship between higher annual patient numbers and increasing number of annual consultations. The data stratified by quartiles of annual patient numbers showed that the slopes of the regression lines decreased for physicians with increasing high patient numbers but, without exception, remained significantly above 1 (95% confidence intervals are not including 1). Pairwise statistical comparisons of these slopes showed significant differences between all four quartiles of patient numbers (Additional file 3: Table 4).

Effect estimates at the service area level show significant positive effects on consultation numbers for higher densities of primary care providers and densities of specialists and regional mortality. Consultations numbers provided by PCP's located in areas with hospitals (Additional file 3: Table 4) were significantly higher. Significantly higher consultation numbers were associated with increasing physician age. General practitioners without specializations provided significantly higher numbers of consultations than physicians certified in family medicine or general internal medicine. No significant differences were observed between physicians with board certification in family medicine or general internal medicine. Increasing numbers of consultations were seen with higher average patient ages and significantly decreasing numbers of consultations were seen with higher proportions of female patient consultations.

Language-stratified data revealed only in Swiss German regions a significant effect of the density of primary care physicians, of specialist density and mortality on consultation numbers. The presence of hospitals providing ambulatory services showed a non-significant effect in all three lingual regions but the slope of the supply curve was significantly higher than 1 in all three regions (Additional file 4: Table 5).

The overall model (model 1) explained 93.2% of the variation in annual consultation frequencies (pseudo-R^2 ^statistics[11]). The analysis of variance components showed that only a small portion of the total variance occurred between physicians within specific health service areas (intraclass correlation coefficient of 0.04). 64% of the explainable variation of the service area level consultation frequency was attributable to the density of PCP and regional mortality accounted for 2.8% of the explainable respective variation. At the physician's level, 93% of the explainable variation was accounted for by the number of patients treated in the same year.

## Discussion

The analyses were based on complete reimbursement data for consultations of the majority of primary care providers with a practice license in Switzerland and were controlled for factors related to professional qualification, physician experience and gender, and for demographic attributes of the patients. Furthermore, we used a spatial model that was directly derived from the demand patterns of the population, which minimized the effect of border crossing patients. The data are therefore representative for Switzerland and we consider the multilevel approach as a more appropriate spatial representation of utilization and the provision of ambulatory care resources than other Swiss analyses based on administrative or political boundaries [14-17].

The calculated annual number of Swiss residents per primary care physician in 2004 was 1250, whereas the respective annual number of patients per physician in our data set turned out to be only 767 and therefore only about 63% of residents are seeing a primary care doctor in a year, which seems low. It is therefore possible that residents have no contacts with a physician or, more likely, are directly consulting with a specialist. However, the true percentage seeing a primary care doctor may be somehow higher as our data lack consultations with overall annual costs below the threshold for reimbursement. These threshold are chosen by patients between the minimum of CHF 300 (≈200€) or up to CHF 1500 (≈1000€) for lower premiums and have to be paid out of pocket.

### Regional effects

Significant correlations between density of PCP's and mortality indicate that areas with high mortality are also characterized by high density of PCP's and high treatment intensity whereas only a weak association was observed between regional mortality and specialist density which is related to the fact that specialist practices are more often located in urban areas characterized with lower mortality than rural areas. However, specialist density was positively associated with higher treatment intensity of PCP's. Mortality also yielded a positive and significant parameter estimate in the modelling procedures implying higher number of consultations in areas with higher mortality. These findings can have multiple interpretations: In high need areas there is high supply of primary care but low supply of specialists care and the results may additionally reflect effects of competition between PCP's and specialists. Yet, the associations between mortality and treatment frequencies of PCP's were not consistent across the three language regions and are also coherent with other observations made earlier in the same project showing that utilisation of primary care and care provided by specialists care also differs across language regions [9]. However, it remains debatable whether mortality rates can be used as a measure of effective medical needs in a health system that is characterized by comprehensive and high density of supply of care; and furthermore, it can also be criticized that a high physician's density is not necessarily related to better medical care at the regional level. Additional data defining regional health status and the respective medical needs are therefore required before such implications can be made.

### Structural attributes of physicians and practices

The high density of supply and the related competition among care providers are factors that are known to induce physician's services[6,7]. Cross sectional results for the impact of GP supply generally indicate that an increase in supply may be associated with an increase in the frequency of consultation[18]. Our data confirm these findings and extend the concept by supplying evidence that higher densities of PCP's, specialists and the availability of out-patient hospital clinics in a given area are associated with higher treatment intensity of PCP's. Depending on the definition of SID[19], it remains debatable whether this observed inducement in competitive areas is related to the fact that PCP's aim to maintain or to increase their income or try to provide more effective and more appropriate care [20], which would imply different levels of treatment quality in areas with different levels of competition. Hospital care may also directly induce services provided by PCP's before and after hospitalization. However, in addition to effect estimates, the statistical procedures also provided variance components which showed that the density of supply has a considerable impact on the regional variance of service volumes. Our data, irrespective of the conceptual problems in defining SID, are therefore consistent with the hypothesis that those non-medical factors related to the competition between PCP's and specialists and between PCP's and out-patient care of hospitals, respectively, affect the quantity of supplied services in primary care.

The observed differences across language regions reflect the decentralized nature of Swiss health care system and are likely attributed to different availabilities of services, to cantonal schemes for reimbursement, and to traditionally anchored behavioural patterns of the respective populations[1,16].

### Characteristics of physicians and patients

The physician level data indicate a disproportionate increase of supplied services irrespective of the size of the number of patients treated during the same time period whereby this disproportionate increase proves to be greater for physicians with smaller numbers of patients. This implies that even in high volume practices no rationing but a paradoxical inducement of consultations occurred. It can thus be assumed that physicians with high annual patient numbers spend less time with their patients and consequently the quality of care may become questionable [21-23]. However, it is also possible that physicians caring for more patients are better and more efficiently organized than their colleagues with fewer patients and that the quality of interactions between physicians and patients thus remains unaffected by high service volumes.

It is unlikely that patient health status in practices with large patient numbers differs substantially from practices with fewer patients, since patient age, one of the dominant factors in general health status, has been accounted for in our analyses. Hence we consider that cases where morbidity negatively influences patient numbers but increases consultation frequencies would not have biased our results.

Our analysis, stratified by quartiles of annual patient numbers, supports the hypothesis that physicians with few patients induce services to a higher degree than their peers with more patients. However, our data do not allow to make a distinct discrimination between the financial incentives to unnecessary induce services and the real need to induce services to meet more intense treatment modalities, as for example in complementary medicine[24], was not possible with our data.

The results of this study can be directly compared with results from Norwegian primary care providers where, in contrast to our findings, proportional relationships between patient numbers and annual consultation frequencies were observed[4]. This discrepancy can likely be attributed to the fact that the majority of Norwegian PCP's are reimbursed on a scheme where capitation is a substantial component of their income[4,25] whereas Swiss physicians (PCP's and specialists) mainly generate their income on a fee-for-service basis. It is therefore reasonable to assume that the disproportionate increase of consultations in the Swiss system is attributable to the scheme of reimbursement in Swiss out-patient care.

The significant effect that physician age had on treatment intensity is certainly related to the positive correlation between the ages of physicians and their patients, reflecting the higher morbidity of elderly patients. Differences in the age structure of female and male physicians and different levels of efficiency of young versus old practitioners may, however, also play a role in this context. Nevertheless, our data on patient characteristics confirmed that higher treatment intensities occur in practices caring for older patients as expected. However, variance component analysis at the practice level indicated that high consultation volumes are mostly driven by high numbers of patients whereas factors related to demographics of patients and physicians accounted for only small fractions of the explainable variance.

The findings from our study demonstrate that competition alone among care providers will not lead to a functioning market. On the contrary, according to Domenighetti[26], SID can be considered a natural phenomenon in systems that guarantee, to a differing degree: a) freedom of prescription b) unrestrained exploitation of new technologies, c) unlimited access to medical training, d) fee-for-service payment for medical activity, and e) unlimited access for the population to medical services without major financial consequences to the individual patient. Yet, the hypothesis of SID in which physicians alter the patient's preferences in their own interest, threatens the economist's basic paradigm about market functioning and undermines the normative implications that underlie economic recommendations about market policy[3]. Many studies conclude that a clinical rather than an economic model of practitioner decision-making provides a more plausible interpretation of the inter-practitioner variation in rates of clinical activity in general practice[27].

Corresponding inter-rater agreement of expert-panels for primary care routines proved at best fair and only 3% of services were judged to be inappropriate[28]. This absence of an unambiguous definition of "appropriate care" makes the identification of SID further complicated.

Nevertheless, to allow a better cost-control, a new national reimbursement scheme based on consultation time and needed logistics was introduced in the year after the study. However, the treatment quality of physicians with high annual numbers of patients and high consultation volumes must remain under scrutiny as the new national 'Tarmed' billing system is still based on a fee-for-service system[29]. Our data may thus be useful as screening tools to highlight areas in which quality should be investigated in greater depth – despite further and future reforms.

### Strength and Limitations

The availability of data from a large majority of Swiss primary care providers who are still under mandatory contracts with health insurers was a strength of the study. The spatial model used in this study yielded service areas in which on average 57% of all primary care services were consumed locally. Therefore, we consider this approach as an appropriate representation of the utilization and provision of primary care within the legal and institutional framework of Swiss health care. Furthermore, the data used in this study cover the overall amount of services as paid for by the basic health insurance companies in Switzerland and hence reflect an accurate basis of the respective reimbursements.

The limitations of the study are mainly related to the fact that only data for physician consultations reimbursed by compulsory health insurance were available. As stated above the volume of consultations is likely to be higher since the first 300 CHF (≈200 €) or more of treatment cost within a year has to be paid out of pocket. Therefore, the patients face different net prices of health services depending on already accrued health expenditures within the current year. This restriction to treatment frequencies did not allow to study the monetary effects of reimbursements as it is done for instance in Delattre and Dormont [30]. Therefore no insight can be provided into the mechanisms that may affect patterns of utilization and provision of care as they are related to financial incentives for consumers and the care providers. Without data on treatment costs it remains difficult to accurately test the hypothesis that demand inducement is associated with the income of care providers. Furthermore, the use of administrative data remains problematic when assessing all aspects of appropriateness and quality of care[31]. The literature, however, provides no clear evidence that higher treatment intensity is necessarily associated with improvements in treatment quality[32].

It may further be argued that spatial variance may be underestimated due to a false assumption of homogeneity in aggregate spatial data[33] and it cannot be excluded that a spatial model based on e.g. administrative boundaries would yield different estimates of rates and variances. However, the closest available level of administrative data in Switzerland would be on the level of the 26 Swiss cantons and comparing 1014 utilisation based service areas with 26 cantons which range from 15'000 to 1 Mio inhabitants will certainly provide considerable differences of estimates. We assume that health service areas are more homogenous than cantons on factors that determine provision and utilisation of health related resources and therefore we consider the observed data and their variation as more relevant than the respective cantonal data.

The data available for the project did not provide estimates of effective medical needs of the population[34] and furthermore, the project cannot account for patient-induced demand, such as that based on a 'decision to consult model';. e.g. it is not possible to differentiate between first consultations initiated mostly by patients and follow-up consultations initiated by doctors as proposed by Day et al[35].

## Conclusion

This study documents high treatment volumes in Swiss primary care and confirms findings from other studies within the same project that various factors affect the patterns of availability and utilization of medical resources. The relationship between density of supply and treatment-incidence[8] and treatment intensity, respectively, and the association between annual number of patients and consultation frequencies provide considerable evidence that the volume of supply in Swiss primary care is not driven by medical needs alone. Therefore effects stimulating higher than necessary treatment volumes that are associated with the system of reimbursing services on a fee-for-service basis can not be excluded. Effects related to the competition for patients between primary care physicians, specialists and out-patient hospital clinics seems to be a further source of increasing treatment intensity in primary care. Additional information is needed to quantify the true medical needs of the population and to determine the quality of services and respective outcomes of provided care by primary care physicians in different practice settings. It thus remains difficult to discriminate between a positive inducement of services to provide primary care of high quality and an inappropriate inducement of services at the expense of treatment quality.

## Competing interests

The authors declare that they have no competing interests.

## Authors' contributions

AB obtained the mandate to perform the study, he performed all statistical analyses and wrote the first draft of the manuscript, PM reviewed and completed the manuscript with reference to economic analyses; BK reviewed and completed the manuscript with reference to all aspects of primary care. All authors read and approved the final manuscript.

## Pre-publication history

The pre-publication history for this paper can be accessed here:



## Supplementary Material

Additional file 1 **Table 2. **Distribution of physicians, number of patients and consultation across language regions in Switzerland. Click here for file

Additional file 2 **Table 3. **Spearman correlation coefficients among health service area and physicians data. Click here for file

Additional file 3 **Table 4.** Effect estimates for annual number of consultations. Click here for file

Additional file 4 **Table 5. **Effect estimates stratified by language region for annual number of consultations (language stratified Model I). Click here for file
