# Supplementary material for: Factors related to treatment intensity in Swiss primary care
Source: BMC Health Serv Res. 2009 Mar 18;9:49. doi: 10.1186/1472-6963-9-49 (PMC2664802; doi:10.1186/1472-6963-9-49)
Supplement: Additional file 1 — Table 2. Distribution of physicians, number of patients and consultation across language regions in Switzerland. [file 1472-6963-9-49-S1.doc]

## Table 2: Distribution of physicians, number of patients and consultation across language regions in Switzerland

| Language region | # primary care physicians (%) | # annual patient number^a^ mean (median) | # annual consultations mean (median) | Treatment intensity^b^ mean (median) |
| --- | --- | --- | --- | --- |
| Swiss German | 4238 (69.62) | 825.22 (812.50) | 3594.79 (3642.00) | 4.56 (4.27) |
| French | 1537 (25.25) | 621.95 (580.00) | 2196.20 (2040.00) | 3.57 (3.46) |
| Italian | 295 (4.85) | 673.61 (673.00) | 3501.16 (3178.00) | 5.11 (5.04) |
| Romansh | 17 (0.28) | 931.65 (924.00) | 3566.12 (3858.00) | 4.23 (4.19) |
| Total of all communities | 6087 (100) | 766.84 (735.00) | 3237.02 (3126.00) | 4.33 (4.08) |

^a^ annual number of patients treated and billed by primary care physicians

^b^ annual number of consultations with primary care physician per patient
