# Supplementary material for: Factors related to treatment intensity in Swiss primary care
Source: BMC Health Serv Res. 2009 Mar 18;9:49. doi: 10.1186/1472-6963-9-49 (PMC2664802; doi:10.1186/1472-6963-9-49)
Supplement: Additional file 2 — Table 3. Spearman correlation coefficients among health service area and physicians data. [file 1472-6963-9-49-S2.doc]

## Table 3: Spearman correlation coefficients among health service area and physicians data

| Level of the data Variable |  |  |  |  |  |
| --- | --- | --- | --- | --- | --- |
| **Health service area** (N = 1004) | PCP density (7.98 PCP’s/10,000 inhabitants) | Specialist density (4.43 Specialists/10,000 inhabitants) | Treatment intensity |  |  |
| Specialists/10,000 inhabitants | 0.186* |  |  |  |  |
| Treatment intensity | 0.037 | 0.119* |  |  |  |
| Mortality  (7.79 deaths/1000 inhabitants) | 0.217* | 0.072* | 0.172* |  |  |
|  |  |  |  |  |  |
| Physician | Age of physician | Average age of patients | Proportion of consultations  for women | Number of consultations | Annual patient number |
| Average age of patients | 0.283^*^ |  |  |  |  |
| Proportion of consultations for women | -0.203^*^ | -0.172^*^ |  |  |  |
| Number of consultations | 0.047^*^ | -0.072^*^ | -0.260^*^ |  |  |
| Annual patient number | -0.063^*^ | -0.246^*^ | -0.225^*^ | 0.881^*^ |  |
| Treatment intensity | 0.216^*^ | 0.281^*^ | -0.104^*^ | 0.409^*^ | 0.017 |

^*^ Significant correlation coefficients
