# Supplementary material for: Factors related to treatment intensity in Swiss primary care
Source: BMC Health Serv Res. 2009 Mar 18;9:49. doi: 10.1186/1472-6963-9-49 (PMC2664802; doi:10.1186/1472-6963-9-49)
Supplement: Additional file 3 — Table 4. Effect estimates for annual number of consultations. [file 1472-6963-9-49-S3.doc]

## Table 4: Effect estimates for annual number of consultations

| Level of data | Variable | Effect estimate | upper / lower 95% confidence limits | | p-value |
| --- | --- | --- | --- | --- | --- |
|  | Constant | -3.438 | -3.768 | -3.109 | <.0001 |
| Service area | PCP/10,000 inhabitants | 0.045 | 0.002 | 0.089 | 0.040 |
|  | Specialists/10,000 inhabitants | 0.031 | 0.014 | 0.048 | <.0001 |
|  | Hospital with out-patient clinics^a^ | 0.046 | 0.005 | 0.088 | 0.028 |
|  | Mortality (# deaths/1000 inhabitants) | 0.079 | 0.028 | 0.130 | 0.002 |
| Physician | Number of patients per year | 1.108 | 1.100 | 1.116 | <.0001 |
| (Model 1) | Physician age | 0.551 | 0.487 | 0.615 | <.0001 |
|  | Physician gender^b^ | 0.061 | 0.031 | 0.092 | <.0.001 |
|  | Professional qualification^c^ |  |  |  |  |
|  | - Practitioner without specialization | 0.081 | 0.050 | 0.112 | <.0.000 |
|  | - General internal medicine | 0.002 | -0.023 | 0.027 | 0.863 |
|  | Proportion of consultations for women | -0.199 | -0.265 | -0.132 | <.0.000 |
|  | Average age of patients | 0.378 | 0.325 | 0.432 | <.0.000 |
|  |  |  |  |  |  |
| Physician | 1. Quartile (P_Q1: ≤ 402 patients) | 1.159 | 1.147 | 1.171 | <.0001 |
| (Model 2^d^) | 2. Quartile (P_Q2: > 402, ≤ 735 patients) | 1.142 | 1.133 | 1.152 | <.0001 |
|  | 3. Quartile (P_Q3: > 735; ≤ 1079 patients) | 1.129 | 1.120 | 1.138 | <.0001 |
|  | 4. Quartile (P_Q4: > 1079 patients) | 1.115 | 1.106 | 1.123 | <.0001 |

^a^ Hospital with out-patient clinics present in the same region, regions without hospital as the reference level.

^b^ Male physicians as the reference level

^c^ Board certification of the Swiss Medical Association for general practice/family medicine, general internal medicine or general practitioner without specialization, family medicine as the reference level

^d^ The remaining cofactors were included in the model but omitted from the table, effect
estimates were very similar to the respective estimates of model 1
