# Supplementary material for: Factors related to treatment intensity in Swiss primary care
Source: BMC Health Serv Res. 2009 Mar 18;9:49. doi: 10.1186/1472-6963-9-49 (PMC2664802; doi:10.1186/1472-6963-9-49)
Supplement: Additional file 4 — Table 5. Effect estimates stratified by language region for annual number of consultations (language stratified Model I). [file 1472-6963-9-49-S4.doc]

## Table 5 Effect estimates stratified by language region for annual number of consultations (language stratified Model I)

| Level of data | Variable | Swiss German | French | Italian |
| --- | --- | --- | --- | --- |
|  | Constant | -2.965^*^ | -4.739^*^ | -3.814^*^ |
| Service area | PCP/10,000 inhabitants | 0.050^*^ | 0.027 | 0.016 |
|  | Specialists/10,000 inhabitants | 0.033^*^ | 0.021 | 0.037 |
|  | Hospital^a^ | 0.017 | 0.038 | 0.130 |
|  | Mortality (# deaths/1000 inhabitants) | 0.089^*^ | 0.072 | 0.011 |
| Physician | Number of patients per year | 1.103^*b^ | 1.113^*b^ | 1.192^*b^ |
|  | Physician age | 0.477^*^ | 0.808^*^ | 0.459^*^ |
|  | Physician gender^c^ | 0.042^*^ | 0.103^*^ | 0.108 |
|  | Professional qualification^d^ |  |  |  |
|  | - Practitioner without specialization | 0.149^*^ | -0.017 | -0.036 |
|  | - General internal medicine | 0.010 | -0.015 | -0.104^*^ |
|  | Proportion of consultations for women | -0.224^*^ | -0.123 | -0.107 |
|  | Average age of patients | 0.339^*^ | 0.439^*^ | 0.543^*^ |

^*^ significant effect

^a^ Hospital providing ambulatory services present the same region, regions without hospital as the reference level.

^b^ 95%confidence limit is not including 1

^c^ Male physicians as the reference level

^d^ Board certifications of the Swiss Medical association for general practice/family medicine, general internal medicine or general practitioner without specialization, family medicine as the reference level
